# Supplementary material for: The Systems Biology Research Tool: evolvable open-source software
Source: BMC Syst Biol. 2008 Jun 29;2:55. doi: 10.1186/1752-0509-2-55 (PMC2446383; doi:10.1186/1752-0509-2-55)
Supplement: Additional file 1 — SBRT Archive. An archive of the current version of the Systems Biology Research Tool. [file 1752-0509-2-55-S1.zip › sbrt-1.4.0/doc/developers_guide/api/sbrt/shell/mng/fba/package-tree.html]

sbrt.shell.mng.fba Class Hierarchy


|  |  |  |
| --- | --- | --- |
| **Overview** \| **Package** \| Class \| Use \| **Tree** \| **Deprecated** \| **Index** \| **Help** | |  |
| **PREV**   **NEXT** | **FRAMES**    **NO FRAMES**     **All Classes** |  |


---


## Hierarchy For Package sbrt.shell.mng.fba

**Package Hierarchies:**: All Packages

---

## Class Hierarchy

- java.lang.Object
  - sbrt.shell.mng.**AbstractProcessManager** (implements sbrt.shell.mng.ProcessManager<K,V>)
    - sbrt.shell.mng.fba.**BiggSbmlReaderManager** (implements sbrt.shell.mng.ShellProcessManager<K,V>)- sbrt.shell.mng.fba.**BiggSbmlTranslaterManager** (implements sbrt.shell.mng.ShellProcessManager<K,V>)- sbrt.shell.mng.fba.**FbaManager** (implements sbrt.shell.mng.KernelProcessManager<K,V>)
          - sbrt.shell.mng.fba.**CatalystDeletionManager**- sbrt.shell.mng.fba.**CvCatalystDeletionManager**- sbrt.shell.mng.fba.**CvFbaOptManager**- sbrt.shell.mng.fba.**CvMsFluxIntervalManager**- sbrt.shell.mng.fba.**CvObjectiveFunctionManager**- sbrt.shell.mng.fba.**CvRevRxnFluxIntervalManager**- sbrt.shell.mng.fba.**CvRxnDeletionManager**- sbrt.shell.mng.fba.**CvSimpleFluxIntervalManager**- sbrt.shell.mng.fba.**ExtremeCurrentManager**- sbrt.shell.mng.fba.**FbaOptManager**- sbrt.shell.mng.fba.**FbaSolverManager**- sbrt.shell.mng.fba.**FluxActivityManager**- sbrt.shell.mng.fba.**FluxCapManager**- sbrt.shell.mng.fba.**FluxPlasticityManager**- sbrt.shell.mng.fba.**InitialPointManager**- sbrt.shell.mng.fba.**IntervalVariationManager**- sbrt.shell.mng.fba.**MsFluxIntervalManager**- sbrt.shell.mng.fba.**MsFluxomeReducerManager**- sbrt.shell.mng.fba.**NetworkInfoManager**- sbrt.shell.mng.fba.**ObjectiveFunctionManager**- sbrt.shell.mng.fba.**RandomConstraintsManager**- sbrt.shell.mng.fba.**RandomEnvManager**- sbrt.shell.mng.fba.**RandomFluxVectorManager**
                                                        - sbrt.shell.mng.fba.**CdHarFluxVectorManager**
                                                        - sbrt.shell.mng.fba.**RandomObjectiveManager**- sbrt.shell.mng.fba.**ReverseRxnFluxIntervalManager**- sbrt.shell.mng.fba.**RxnDeletionManager**- sbrt.shell.mng.fba.**SimpleFluxIntervalManager**- sbrt.shell.mng.fba.**SlpCycleIdManager**- sbrt.shell.mng.fba.**StoichVariationManager**- sbrt.shell.mng.fba.**SvCdHarFluxVectorManager**- sbrt.shell.mng.fba.**SvInitialPointManager**- sbrt.shell.mng.fba.**WwFluxomeReducerManager**
          - sbrt.shell.mng.fba.**MetatoolFileWriterManager** (implements sbrt.shell.mng.ShellProcessManager<K,V>)- sbrt.shell.mng.fba.**MmaNotebookManager**- sbrt.shell.mng.fba.**PalssonSbmlReaderManager** (implements sbrt.shell.mng.ShellProcessManager<K,V>)- sbrt.shell.mng.fba.**PalssonSbmlTranslaterManager** (implements sbrt.shell.mng.ShellProcessManager<K,V>)- sbrt.shell.mng.fba.**RxnRemovalManager**- sbrt.shell.mng.fba.**SbmlWriterManager** (implements sbrt.shell.mng.ShellProcessManager<K,V>)- sbrt.shell.mng.fba.**SimpleRxnFileTransManager** (implements sbrt.shell.mng.ShellProcessManager<K,V>)
    - sbrt.kernel.io.**AbstractResultsManager**<R> (implements sbrt.kernel.io.ResultsManager<T>)
      - sbrt.shell.mng.fba.**ExtremeCurrentManager.Rm**

---


|  |  |  |
| --- | --- | --- |
| **Overview** \| **Package** \| Class \| Use \| **Tree** \| **Deprecated** \| **Index** \| **Help** | |  |
| **PREV**   **NEXT** | **FRAMES**    **NO FRAMES**     **All Classes** |  |


---
